# Full-Length Transcriptome of Testis and Ovary Provides Insights into Alternative Splicing During Gonadal Development in *Litopenaeus vannamei*

**DOI:** 10.3390/ijms26125863

**Published:** 2025-06-19

**Authors:** Youyan Wang, Yang Yu, Yue Wang, Fuhua Li

**Affiliations:** 1 School of Marine Science and Engineering, Qingdao Agricultural University, Qingdao 266109, China; 2 State Key Laboratory of Breeding Biotechnology and Sustainable Aquaculture, Institute of Oceanology, Chinese Academy of Sciences, Qingdao 266000, Chinafhli@qdio.ac.cn (F.L.); 3 Laboratory for Marine Biology and Biotechnology, Qingdao Marine Science and Technology Center, Qingdao 266071, China

**Keywords:** sexual differentiation, PacBio Iso-Seq, AS events, gonads, shrimp

## Abstract

The Pacific white shrimp, *Litopenaeus vannamei* (*L. vannamei*), is an important aquaculture species, yet the molecular mechanisms underlying its sex differentiation and gonadal development remain poorly understood. A deeper understanding of these processes is critical for advancing broodstock quality and enabling unisex breeding strategies. While previous studies have focused on gene expression differences between females and males, structural differences in transcriptomic regulation between sexes have been largely overlooked. Here, we present a comprehensive full-length transcriptome analysis of *L. vannamei* testis and ovary, identifying 830 and 690 novel genes, respectively, and over 6000 new isoforms. Notably, we discovered extensive alternative splicing (AS) events, with the cartilage oligomeric matrix protein-like gene exhibiting over 300 AS isoforms in the ovary compared to only 2 in the testis, suggesting a potential role in ovarian development. Furthermore, sex-determining genes such as *Fem-1a*, *Fem-1c*, and *Sxl* were found to produce AS isoforms exclusively in ovarian tissue. We also identified three germ cell development-associated genes—*MAD2-like*, *RAD51-like*, and *Su(dx)-like*—that undergo distinct AS events in gonadal tissues, leading to sex-specific structural domain alterations. These findings highlight the complexity of AS-mediated post-transcriptional regulation in *L. vannamei* and provide novel insights into the molecular mechanisms governing sex differentiation and gonadal development.

## 1. Introduction

The Pacific white shrimp *Litopenaeus vannamei* (*L. vannamei*) has become the world-leading shrimp aquaculture species due to its fast growth, adaptability to the environment, and resistance to disease [1]. According to the FAO, its production in 2022 was estimated to be about 6.8 million tons [2]. A sufficient supply of high-quality broodstock is fundamental to the development of the shrimp industry, and research on gonadal development provides crucial guidance for breeding superior broodstock [3]. Moreover, the growth traits of *L. vannamei* are significantly sexually dimorphic [4], with females being larger than males at sexual maturity [5], and studies on gonadal differentiation can help to achieve monosex culture through sex control techniques [6]. However, most of the current research on *L. vannamei* has focused on immunity [7,8] and growth [9,10], and there is a relative lack of research on its gonadal development [11]. Studying the genetic regulation of gonadal development is crucial for elucidating sex-specific differences and for advancing strategies to promote gonadal development artificially and enable unisexual breeding [12,13].

Identifying genes associated with gonadal differentiation and development is fundamental to understanding gonadal differentiation, and previous studies have reported several genes related to gonadal development in crustaceans [14]. Using RNAi technology, it was found that *EcR* could promote ovarian development in *Scylla paramamosain* by regulating the expression of *Vg* [15]. Moreover, *SoxB2* gene plays an important role in the maturation of sperm cell nuclei in *Eriocheir sinensis* [16]. The *IAG* is a master switch gene in sex differentiation and its expression induces masculinization, whereas deletion of AG or lack of IAG expression leads to feminization [17]. Most of the current research on sexual dimorphism has focused on the gene expression difference between males and females, and there is a relative scarcity of research on AS in aquaculture species. AS implies that the same pre-mRNA produces several mRNA splicing isoforms with different sequences or structures through different splicing methods, encoding proteins with different functions and subcellular localization [18,19]. AS is a common phenomenon in eukaryotic gene expression and is a key feature of gene evolution, as it increases proteomic diversity without increasing the number of genes [20]. Since females and males largely share the same genome, sex-biased AS can be used as an alternative mechanism to produce sexually dimorphic traits relative to sex-biased gene expression [21]. The most well-known AS regulation is the somatic sex-determination pathway in *Drosophila* [22,23,24]. The sex-lethal (*SXL*), transformer (*TRA*), and transformer 2 (*TRA2*) form a multi-level splicing cascade that regulates the downstream targets male-specific lethal-2, fruitless, and doublesex for gender-specific splicing [25,26,27]. AS events have been reported to be involved in several biological processes in *L. vannamei*. For example, genome-wide analysis of AS events in *L. vannamei* revealed that AS events may respond to various external environmental stresses [28]. Transcriptome analysis reveals that AS may play a role in transcriptional regulation in response to heat stress [29]. The Lactate dehydrogenase (*LDH*) produces two distinct *LDH* transcripts by AS to respond to hypoxic metabolic regulation during hypoxia [30]. AS has also been reported to be involved in the response to acute alkalinity stress in *L. vannamei* [31]. In addition, AS also plays a regulatory function in immunity [32]. However, relatively little is known about AS regulation during gonadal development in *L. vannamei*. The study of AS regulation contributes to a deeper understanding of the mechanisms of sex differentiation in *L. vannamei*.

Isoform sequencing (Iso-Seq) using the PacBio platform offers long read lengths that enable the reconstruction of full-length mRNA sequences, including poly(A) tails, 5′ and 3′ untranslated regions (UTRs), and intact splicing events. This approach facilitates the discovery of novel genes and the identification of complex alternative splicing isoforms with high accuracy. There are two major applications of ISO-seq in genomic research; the first one is to identify alternative isoforms, alternative polyadenylation, and non-coding RNA (ncRNA) [33], and the second one is used for genome annotation and the identification of new genes and new isoforms [34]. Iso-Seq has now been extensively applied to a range of aquatic species, including *Scophthalmus maximus* [35], *Penaeus monodon* [36], *Artemia franciscana* [37], and *Exopalaemon carinicauda* [38]. By providing comprehensive transcriptome profiles, Iso-seq facilitates a deeper understanding of gene expression dynamics and regulatory mechanisms in these organisms.

In this study, we utilized PacBio SMRT sequencing to generate a high-quality, full-length transcriptome of the testis and ovary in *L. vannamei*. The resulting data enabled comprehensive analyses, including functional annotation of transcripts, alternative splicing prediction, long non-coding RNA (LncRNA) identification, and transcription factor prediction. We conducted comparative analyses of gene expression profiles and alternative splicing isoforms between the testis and ovary. This research provides critical insights and a valuable resource for elucidating the molecular mechanisms underlying sex differentiation in *L. vannamei.*

## 2. Results

### 2.1. Overview of PacBio Iso-Seq Sequencing

**Figure 1:**
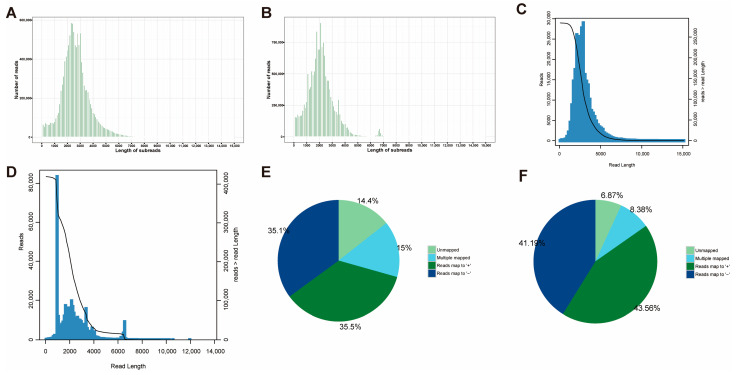
Summary of Iso-seq data. (**A**,**B**) Length distribution of subreads; the horizontal coordinate indicates the length of the subreads, the vertical coordinate is the number of subreads, (**A**) denotes the length distribution of subreads for testis, (**B**) denotes the length distribution of subreads for ovary. (**C**,**D**) FLNC length distribution map; the *x*-axis indicates the length of the FLNC reads, the left *Y*-axis is a bar graph coordinate indicating the number of reads in a range of lengths (*X*-axis), the *Y*-axis on the right is the plot coordinates indicating the number of reads whose length is greater than a certain *X*-axis value, (**C**) denotes the FLNC length profile of testis, and (**D**) denotes the ovary. (**E**,**F**) The percentage of high-quality isoforms mapped to the reference genome; (**E**) denotes the result for testis and (**F**) denotes the result for ovary.

A total of 15,597,803 and 12,098,870 subreads were obtained for the ovary and testis samples, respectively. The average lengths of the subreads were 2714 bp (testis) and 2180 bp (ovary) (Figure 1A,B). A total of 483,391 (testis) and 730,978 (ovary) circular consensus sequences (CCSs) were obtained. All CCSs were further classified into 284,013 (62.75% of testis CCS) and 419,324 (63.31% of ovary CCS) full-length non-chimeric (FLNC) sequences (Figure 1C,D). FLNC reads were clustered into 18,904 (98% of testis) and 18,564 (97.9% of ovary) high-quality isoforms (HQ isoforms) (with prediction accuracies of ≥0.99). High-quality (HQ) isoforms were mapped to the reference genome (RefSeq: GCF_003789085.1) using Minimap2, in which 13,349 (70.61% of testis) and 15,734 (84.76% of ovary) HQ isoforms were uniquely mapped, while 2838 (15.01% of testis) and 1555 (8.38% of ovary) HQ isoforms were multiple mapped, leaving 2717 (14.37% of testis) and 1275 (6.87% of ovary) unmapped (Figure 1E,F). The results were clustered and de-redundant to obtain unique transcripts, counting the number of known genes as well as the number of new genes in each sample. A total of 4392 and 3942 genes were mapped to the testis and ovary, respectively. Among them, a total of 3983 (testis) and 2834 (ovary) known isoforms were obtained, 830 (testis) and 690 (ovary) novel isoforms were discovered, and 6017 (testis) and 7124 (ovary) new isoforms were found (Table 1).

**Figure 2:**
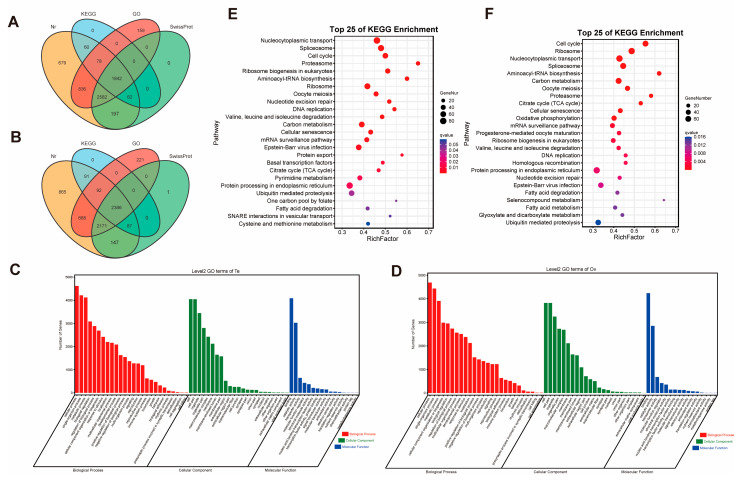
Function annotation and classification of isoforms. (**A**) Venn diagrams of annotated isoforms in testis. (**B**) Venn diagrams of annotated isoforms in ovary. (**C**) Annotation of GO functional classification of isoforms in testis. (**D**) Annotation of GO functional classification of isoforms in ovary. (**E**) KEGG pathway enriched for genes in testis. (**F**) KEGG pathway enriched for genes in ovary.

Isoforms were functionally annotated using the Swiss-Prot, Gene Ontology (GO), non-redundant protein (Nr), and Kyoto Encyclopedia of Genes and Genomes (KEGG) databases. In the ovary, a total of 6507 isoforms were annotated in Nr, 2656 in KEGG, 4792 in Swiss-Prot, and 5538 in GO. Similarly, in the testis, 6336 isoforms were annotated in Nr, 2042 in KEGG, 4683 in Swiss-Prot, and 5497 in GO. Across all databases, 6729 isoforms in the ovary and 6495 in the testis were annotated in at least one database, while 2386 and 1842 isoforms, respectively, were assigned annotations in all four databases (Figure 2A,B). In GO annotation, the majority of isoforms were enriched in the molecular functions of binding (45% for ovary, 43% for testis) and catalytic activity (31% for ovary, 32% for testis), the biological process categories of cellular process (11% for ovary, 11% for testis) and single-organism process (11% for ovary, 10% for testis), and the cellular component categories of cell (15% for ovary, 17% for testis) and cell part (ovary of 15%, testis of 17%) (Figure 2C,D). In the KEGG classification of the testis, genes were significantly enriched in nucleocytoplasmic transport, spliceosome, cell cycle, proteasome, and ribosome biogenesis in eukaryotes pathways (Figure 2E). In the KEGG classification of the ovary, genes were significantly enriched in pathways such as cell cycle, ribosome, nucleocytoplasmic transport, spliceosome, and aminoacyl-tRNA biosynthesis (Figure 2F).

### 2.2. Comparison of Genes Between Ovary and Testis

**Figure 3:**
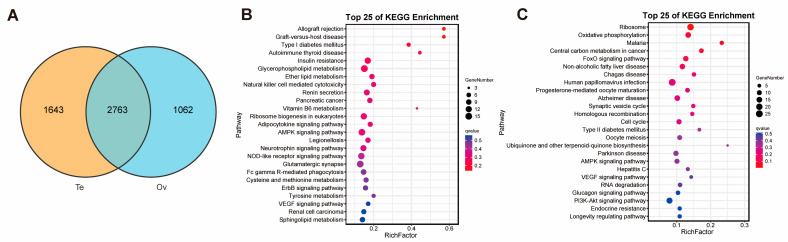
Gene statistics and KEGG enrichment in testis and ovary. (**A**) Venn diagrams for genes in the testis and ovary. (**B**) KEGG pathways enriched for genes specific to the testis. (**C**) KEGG pathways enriched for genes specific to the ovary.

Among the genes mapped to the reference genome for the ovary and testis, 2763 were shared between the two gonadal tissues (Figure 3A). A total of 1062 genes were specifically identified in the ovary, enriched in pathways such as ribosome, oxidative phosphorylation, and malaria (Figure 3C). In contrast, 1643 genes were uniquely identified in the testis, with enrichment in pathways including allograft rejection, graft-versus-host disease, and type I diabetes mellitus (Figure 3B).

### 2.3. Alternative Splicing Analysis

**Figure 4:**
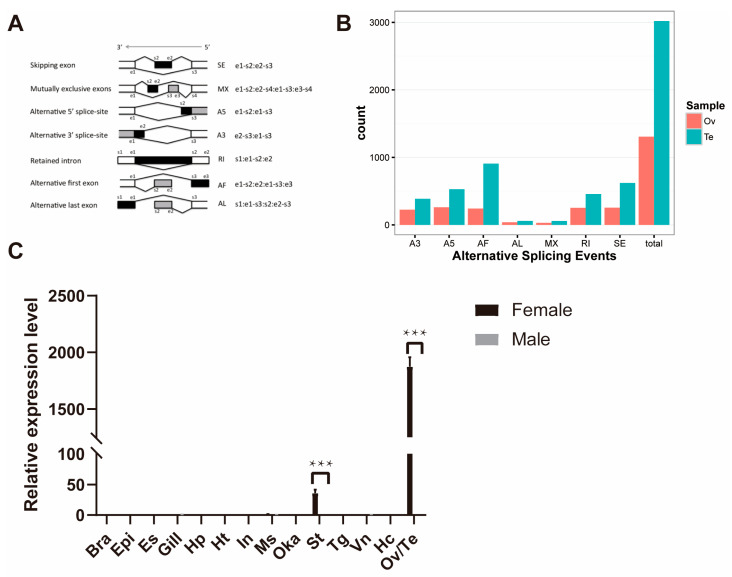
Alternative splicing analysis. (**A**) Classification of alternative splicing events. (**B**) Distribution of the number of alternative splicing events. (**C**) Expression pattern of the gene LOC113825377 (NCBI Gene ID: 113825377) in different tissues of adult shrimp *L. vannamei*. Adult different tissues: Bra (brain), Epi (epidermis), Es (eye stalk), Gill (gill), Hp (hepatopancreas), Ht (heart), In (intestine), Ms (muscle), Oka (lymphoid organ), St (stomach), Tg (thoracic ganglion), Vn (ventral nerve), Hc (hemocyte), Ov (ovary), Te (testis). *** indicates *p* < 0.001.

SUPPA [39] was used to ascertain the frequencies of seven major AS types (skipping exon, SE; alternative 5′ splice sites, A5; alternative 3′ splice sites, A3; mutually exclusive exons, MX; retained intron, RI; alternative first exons, AF; alternative last exons, AL) in the testis and ovary iso-Seq data (Figure 4A). In total, 3019 AS events were identified in the testis. The most abundant of these was AF, which accounted for 30.08% of all AS events in the testis. In total, 1307 AS events were identified in the ovary. In contrast to the testis, the ovary was most common in A5, accounting for 19.97% of all AS events in the ovary. However, the four types of AS events, SE, RI, AF, and A3, accounted for a similar and close to A5 proportion of all AS events in the ovary. In both tissues, the fewest AS events were identified in AL (1.95% of testis and 2.98% of ovary) and MX (1.92% of testis and 2.37% of ovary) (Figure 4B).

In the ovary, 93 genes were present in more than 10 AS isoforms. In the testis, 67 genes were present with more than 10 isoforms of AS. Interestingly, one gene (LOC113825377) annotated as cartilage oligomeric matrix protein-like was found to have more than 100 AS isoforms in the ovary (Supplementary Figure S1). The annotations for LOC113821441 (NCBI Gene ID: 113821441), LOC113815815 (NCBI Gene ID: 113815815), LOC113821443 (NCBI Gene ID: 113821443), LOC113824160 (NCBI Gene ID: 113824160), LOC113830308 (NCBI Gene ID: 113830308), LOC113823139 (NCBI Gene ID: 113823139), and LOC113823118 (NCBI Gene ID: 113823118) are similarly cartilage oligomeric matrix protein-like. This class of genes has up to 300 AS isoforms in the ovary, yet only 2 AS isoforms in the testis. LOC113825377 shows significant expression differences in adult gonads as well as in other different tissues and is almost ovary-specific (Figure 4C). This suggests that the gene plays a role in the development and maintenance of the ovary.

### 2.4. Annotation and Comparison of AS in Ovary and Testis

Genes undergoing AS in the ovary and testis were functionally annotated using the KEGG database (Supplementary Figure S2). These alternative splicing genes were assigned to 322 pathways in the ovary and 330 pathways in the testis. Notably, in the ovary, 26, 9, 27, 29, 15, 48, and 17 alternative splicing genes were associated with the MAPK signaling pathway (ko04010), GnRH signaling pathway (ko04912), progesterone-mediated oocyte maturation (ko04914), focal adhesion (ko04510), calcium signaling pathway (ko04020), ubiquitin-mediated proteolysis (ko04120), and wnt signaling pathway (ko04310), respectively. These pathways were involved in oogenesis, spermatogenesis, and gonadal maturation. In the testis, 36, 12, 21, 31, 11, 50, and 19 AS genes were assigned to the same KEGG pathway as the total genes of the testis described above.

A total of 28 genes associated with gonadal differentiation and germ cell development were identified containing different AS events in the testis and ovary (Supplementary Table S1). Among these genes, the cyclin-dependent kinase 1-like (*CDK1*), which is associated with germ cell meiosis [40], exhibits two AS isoforms in both the testis and ovary, one of which is shared between the two tissues. The ubiquitin-conjugating enzyme E2 J1-like (*UBE2J1-like*), a gene involved in the process of spermatogenesis, has three AS isoforms in both the testis and ovary, one of which is shared between the two tissues. The gene G1/S-specific cyclin-E1-like (*CCNE-like*), which is associated with gonadal development, displays eight forms of AS isoforms in the ovary and only one form of AS isoform in the testis. In addition, the gene Calmodulin (*CALM*), which is involved in the process of oocyte development [41], exhibits a large number of AS isoforms in both the testis and ovary, with 11 forms in the ovary and 10 forms in the testis. The Suppressor of deltex-like (*Su(dx)-like*) gene, which is involved in the process of oogenesis, has three AS isoforms in the ovary and two in the testis.

**Figure 5:**
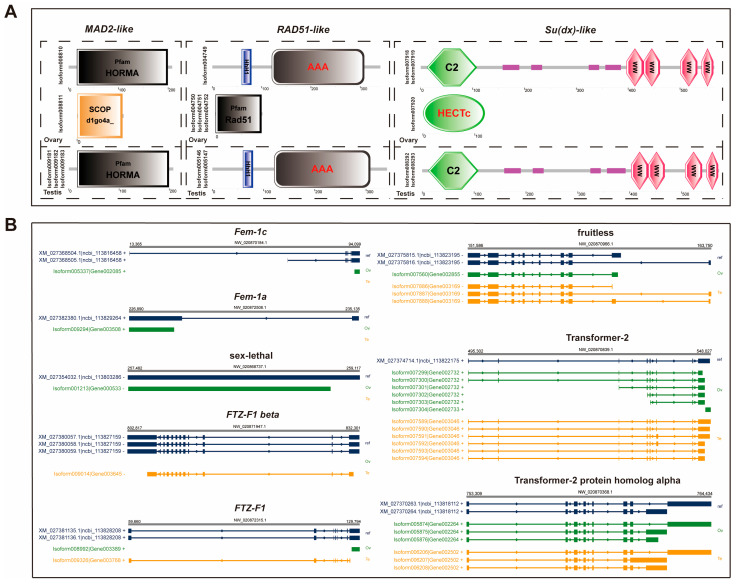
Alternative splicing isoform analysis. (**A**) Structural domains of different transcripts of *MAD2-like*, *RAD51-like*, and *Su(dx)-like*. (**B**) Alternative splicing analysis of known sex-determining and differential genes.

AS isoforms of the above genes were subjected to structural domain prediction for further analysis. We found that AS events led to significant differences in the structural domains of mitotic arrest deficient 2-like (*MAD2-like*), DNA repair protein RAD51-like (*RAD51-like*), and *Su(dx)-like* genes and the testis and ovary showed significant differences. As shown in Figure 5A, these three genes are predicted to possess two structural domain forms in the ovary, while only a single form is observed in the testis. The novel structural domains resulting from AS may contribute to additional functional roles for these genes in the ovary.

### 2.5. AS Analysis of the Known Sex-Determination and Differentiation Genes

Analysis of Iso-Seq data from the ovary and testis enabled the examination of known sex-determination and differentiation genes, revealing their AS events (Figure 5B). In the ovary, the genes *fem-1a* and *fem-1c* were identified, along with novel isoforms. Additionally, a new isoform of the sex-lethal homolog was detected. In the testis, *FTZ-F1 beta* was identified, and one novel isoform was characterized. Furthermore, new isoforms for fruitless, transformer-2, transformer-2 protein homolog alpha, and *FTZ-F1* were found in both the ovary and testis. Notably, transformer-2 exhibited the greatest number of newly discovered isoforms among these sex-determination and differentiation genes, with six isoforms identified in both the ovary and testis. Similarly, transformer-2 protein homolog alpha demonstrated three novel isoforms in each tissue.

### 2.6. Verification of the ISO-Seq Results

**Figure 6:**
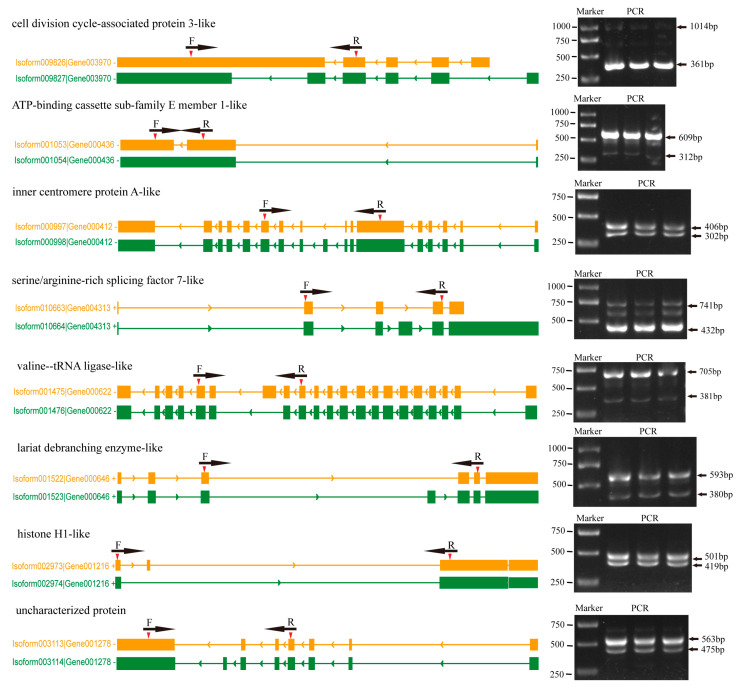
Validation of selective splicing (AS) events. Gene names and transcript IDs are shown sequentially on the left side of the gene model. The exons of the different transcripts are indicated by yellow or green squares, respectively, and red arrows indicate the position of the PCR primers (F for forward and R for reverse). Introns are indicated by lines. In agarose gel images, arrows indicate PCR products generated from different transcripts.

To verify the accuracy of the isoforms identified by Iso-Seq, 10 genes containing AS events were randomly selected and the presence of the different isoforms was verified by PCR and gel electrophoresis. Primers were designed in the overlapping regions of different transcripts of the same gene. The experimental results showed that 8 out of 10 genes were successfully validated. The sizes of the amplified products were consistent with the target fragments predicted by Iso-Seq, and the Sanger sequencing of these amplified products proved to be the same as the Iso-seq sequencing results, which confirmed the confidence of the Iso-Seq data (Figure 6).

## 3. Discussion

In this study, we utilized PacBio Iso-Seq to uncover the transcriptome of gonadal tissue in *L. vannamei*. A total of 690 and 830 novel genes were identified in the ovary and testis, and more than 6000 new isoforms were identified; this valuable information is useful in improving genome annotation and deepening our understanding of gene diversity of *L. vannamei*. Our findings highlight the critical role of AS in the development of ovarian and testicular tissues and some ASs are specific in the ovary or testis, which means they may be the key genes in gonadal development. These results contribute valuable resources and insights for elucidating the molecular mechanisms of gonadal development and sex differentiation in *L. vannamei*.

With the development of sequencing technology, single-molecule real-time (SMRT) sequencing has been widely applied in aquatic animal research. This powerful technology has facilitated studies on a variety of biological processes, including immune response [35,42], growth [43], and the discovery of genes related to reproduction and development [44]. Based on PacBio Iso-Seq and Illumina RNA-Seq technologies, 39 potential sex-determining genes, including the *DMRT1* and *Sad* genes, were identified in *Artemia* [37]. For *E. carinicauda*, Iso-seq and RNA-Seq sequencing identified important genes and pathways involved in ovarian maturation [38]. Similarly, Iso-Seq has been employed to identify differentially expressed genes related to gonadal development and maturation in *S. paramamosain* [45]. All these reflect the important role of SMRT sequencing in sex differentiation studies. In our study, we conducted a comprehensive analysis of AS events in ovarian and testicular tissues and identified numerous genes exhibiting diverse isoforms. Among these, we found several genes annotated as cartilage oligomeric matrix protein-like (*COMP-like*), and this class of genes has up to 300 AS isoforms. It is the class of genes that contains the highest number of AS isoforms. In mice, *COMP* was shown to be involved in ovarian follicle development [46]. In nesting chickens, the gene has been reported to possibly play a role in ovarian development [47]. In our study, the expression pattern of *COMP-like* in different tissues of adult shrimp showed ovary-specific expression. This suggests that the gene may be functionally similar as in mammals. It is essential for ovarian development and maintenance of function.

Gonad differentiation and development is a complex process influenced by a variety of factors, including genetic, environmental, and endocrine regulation. It includes sex differentiation, gonad formation, and gametogenesis [48]. Several genes have been proved to be involved in these processes; some are important for testis development such as *Dmrt1* [49], Doublesex [50], *Fru* [51], *sox* family [52], and *IAG* [53], while genes such as *Sxl* [54], *Fem-1* [55], *Tra2* [56], *Ftz-f1* [57], *Foxl2* [58], and Vitellogenin [59] are important for ovary development. In this study, we observed that some of these genes were specifically detected in the corresponding gonadal tissues. Importantly, we also identified several novel isoforms that may play critical roles in development. In the analysis of AS events in the transcriptome, two *Ftz-f1*, one *Fru*, and two *Tra* were obtained, and we identified new AS isoforms of these three genes in the testis and ovary. This enriches the genetic diversity of *L. vannamei*. *Fem-1a*, *Fem-1c*, and *Sxl* have been shown to be involved in ovarian development in other species [60,61]. In our study, AS events were also detected in these three genes. Interestingly, we detected AS isoforms of these genes only in the ovary, which may be related to the function of this gene during ovarian development.

In addition, we found that genes associated with ovarian development showed a higher form of AS. Much of the research now focuses on studying the differential expression of these genes, rather than the AS events that occur during this process. In the present study, we focused on AS events of the three genes that led to different changes in their structural domains in the testis and ovary. The role of *MAD2* in gonadal development is mainly characterized by its influence on the meiotic maturation process of oocytes [62]. Chromosome segregation and spindle checkpoint control are affected by altered *MAD2* levels in mice [63]. *RAD51* likewise plays a role in meiosis. *RAD51* has been reported to be expressed in the gonads of *M. rosenbergii*, in the ovary, mainly in stage 2-3 oocytes, and in the testis, the gene is enriched only in spermatogonia [64]. *Su(dx)* regulates interfollicular stalk formation, egg chamber separation, and germline cyst envelopment by the follicle stem cells in *D. melanogaster* [65]. The above studies suggest that these three genes play a role in meiosis or oogenesis. Our results provide reference information for future experimental studies.

## 4. Materials and Methods

### 4.1. Animal Material and RNA Sample Preparation

Three mature male and three female individuals were selected, with average body weights of 42.5 g for males and 47.9 g for females. The ovaries from all female individuals were confirmed to be at developmental stage III. Gonadal tissues were dissected from three females and three males, with ovaries (Ov) and testes (Te) pooled separately. The pooled samples were immediately snap-frozen in liquid nitrogen to preserve RNA integrity. Total RNA of the ovary and testis samples was extracted using RNAiso Plus (Takara, Kyoto, Japan) according to the manufacturer’s instructions. The quality and concentration of RNA samples were determined by 1% agarose gel electrophoresis and NanoDrop 2000 spectrophotometer (Thermo Fisher Scientific, Waltham, MA, USA).

### 4.2. PacBio Iso-Seq Sequencing

PolyA-containing mRNA was enriched by magnetic beads with Oligo(dT) and then reverse transcribed to cDNA using a Clontech SMARTer PCR cDNA Synthesis Kit (Takara, Kyoto, Japan). PCR cycle optimization was used to determine the optimal number of amplification cycles for downstream large-scale PCR reactions. The optimized number of cycles was then used to generate double-stranded cDNA. In addition, >4 kb size selection was performed using the BluePippin^TM^ Size-Selection System and mixed homogeneously with non-size-selected cDNA. Large-scale PCR was then performed to construct the next SMRTbell library. The cDNA was DNA damage repaired, end repaired, and ligated to the sequencing junction. The SMRTbell template was annealed to the sequencing primer and combined with polymerase, and qualified libraries were sequenced on the PacBio RS II platform, and sequencing was performed by Tianjin Biochip Corporation (Tianjin, China). All raw reads were deposited on the NCBI Sequence Reading Archive (SRA) website (SRR33113160 and SRR33113161).

### 4.3. PacBio Data Processing

Raw data from cDNA libraries were processed to obtain subreads using the SMRT Link v8.0.0 pipeline (default parameter) supported by Pacific Biosciences [66], and circular consensus sequence was obtained from subreads BAM files, which were classified into full-length non-chimeric (FL), non-full-length (nFL), chimeric, and short reads based on the presence or absence of 5′primer, 3′primer, and polyA structures. Full-length non-chimeric (FLNC) reads were clustered using Minimap2 v2.24 software (default parameter) to obtain consistent sequences. High-quality sequences were mapped to the published reference genome (RefSeq: GCF_003789085.1) [67] using Minimap2 v2.24 (default parameter) [68].

### 4.4. Functional Annotation of Transcripts

The open reading frames (ORFs) were detected by using ANGEL v2.24 [69] software (default parameter) for the isoform sequences to obtain the coding sequences (CDS), protein sequences, and UTR sequences. Functional annotation information of the new isoforms was obtained based on the Nr, KEGG, and Swiss-Prot databases. GO annotation was analyzed by fasterGO v2.0.9.147 software (default parameter) [70] with the Nr annotation results of isoforms and the top 20 isoforms with the highest scores and no less than 33 high-scoring segment pair (HSPs) hits were selected to conduct fasterGO v2.0.9.147 analysis. Functional classification of isoforms was performed using WEGO v2.0 software (default parameter) [71]. The significance threshold was set at *p* value < 0.05.

### 4.5. AS Analysis

AS events in the above non-redundant transcripts were identified using the SUPPA v2.0 [39] tool with the boundary set to the default value. Seven major types of AS events, namely ES, RI, A5, A3, MX, AF, and AL, were classified and counted, and were compared between different samples. Genes with AS events were functionally enriched using the KEGG database. The significance threshold was set at *p* value < 0.05. Structural domain prediction of the screened genes was performed using the SMART website (https://smart.embl.de/smart/set_mode.cgi?NORMAL=1, accessed on 9 December 2024).

### 4.6. Validation Experiments

The expression of the identified genes in different tissues of adult shrimp was analyzed by RT-qPCR. The following tissues were taken from 9 female and 9 male shrimps: brain, epidermis, eye stalk, gill, hepatopancreas, heart, intestine, muscle, lymphoid organ, stomach, thoracic ganglion, ventral nerve, hemocyte, ovary, and testis. Different tissues from each of three individuals of the same sex were mixed separately. In total, three sets of female and male tissues were obtained. RNA was extracted from these three sets of male and female tissues. Total RNA was reverse transcribed to cDNA using the PrimeScript™ RT Reagent Kit containing gDNA Eraser (Takara, Kyoto, Japan) according to the manufacturer’s instructions and diluted 30-fold with nuclease-free water. Primers (Supplementary Table S2) for RT-qPCR were designed using the Primer 3 website (https://www.primer3plus.com/, accessed on 9 January 2025). Expression levels were normalized using *18s* rRNA and *β-actin* as an internal control. RT-qPCR was performed using THUNDERBIRD qPCR Mix (TOYOBO, Osaka, Japan) on an Eppendorf Mastercycler ep realplex (Eppendorf, Hamburg, Germany) according to the manufacturer’s instructions. The amplification steps were as follows: 2 min at 95 °C; then 40 cycles of 15 s at 95 °C, 15 s at 55 °C, and 30 s at 72 °C; followed by a melting curve. Relative expression was calculated using the 2^−ΔΔCT^ method.

The AS events of the genes were verified by conventional PCR and gel electrophoresis. Total RNA was extracted from three sets of gonadal tissues, with each set comprising three biological replicates. Total RNA was reverse transcribed to cDNA using the PrimeScript™ RT Reagent Kit containing gDNA Eraser (Takara, Kyoto, Japan) according to the manufacturer’s instructions. Conventional PCR was performed using 30× diluted cDNA as a template with ExTaq enzyme (Takara, Kyoto, Japan). Primers were designed using the Primer3 website (Supplementary Table S2). PCR conditions were as follows: 5 min at 94 °C, followed by 35 cycles of 30 s at 94 °C, 30 s at Tm, 72 °C for a time period that depends on the product sizes, and 10 min at 72 °C. PCR products were detected using 1% agarose gel electrophoresis, and the target bands were sequenced using Sanger sequencing.

## 5. Conclusions

In this study, we analyzed 10,830 (testis) and 10,648 (ovary) transcripts from the testis and ovary of *L. vannamei* using SMRT sequencing. A total of 830 novel isoforms were identified in the testis, and 690 in the ovary. Additionally, 3019 and 1307 AS events were identified in the testis and ovary, respectively, enriching the genome annotation of *L. vannamei*. In these AS events, known sex-determining and differentiation genes were identified as having new isoforms. In addition to this, we have found that some genes associated with oocyte meiosis undergo different AS events in the testis and ovary, generating different isoforms, as well as leading to differences in the gene structural domains of these genes between the testis and ovary. Overall, our findings provide new insights into gonadal differentiation and development and post-transcriptional regulation.

## Figures and Tables

**Table 1:** Overview of results of PacBio Iso-seq.

Subjects	Data	Number (%)/Length (bp)	
		Testis	Ovary
Subreads	Total base (bp)	32,844,540,629	34,014,931,007
	Subreads number	12,098,870	15,597,803
	Average length (bp)	2714	2180
	N50 (bp)	2953	2443
Number of CCSs	Number of CCS reads	483,391	730,978
	Full-length non-chimeric, FLNC	284,013	419,324
	Mean full-length non-concatemer read length (bp)	2946	2225
HQ isoform mapped to genome	Uniquely mapped	13,349 (70.61%)	15,734 (84.76%)
	Multiple mapped	2838 (15.01%)	1555 (8.38%)
	Unmapped	2717 (14.37%)	1275 (6.87%)
Reference transcripts	All mapped genes	4392	3942
	All mapped isoforms	10,830	10,648
	Known isoforms	3983	2834
	Novel isoforms	830	690
	New isoforms	6017	7124

## Data Availability

The datasets presented in this study can be found in online repositories. The names of the repository/repositories and accession number(s) can be found in the article.

## Supplementary Materials

The following supporting information can be downloaded at: https://www.mdpi.com/article/10.3390/ijms26125863/s1.
