# Supplementary material for: Full-Length Transcriptome of Testis and Ovary Provides Insights into Alternative Splicing During Gonadal Development in Litopenaeus vannamei
Source: Int J Mol Sci. 2025 Jun 19;26(12):5863. doi: 10.3390/ijms26125863 (PMC12192809; doi:10.3390/ijms26125863)
Supplement: Supplementary file 1 [file ijms-26-05863-s001.zip › Supplementary Figure.pdf]

Figure S1: Comparative structural map of AS isoforms of gene ncbi_113825377 in gonadal tissues.

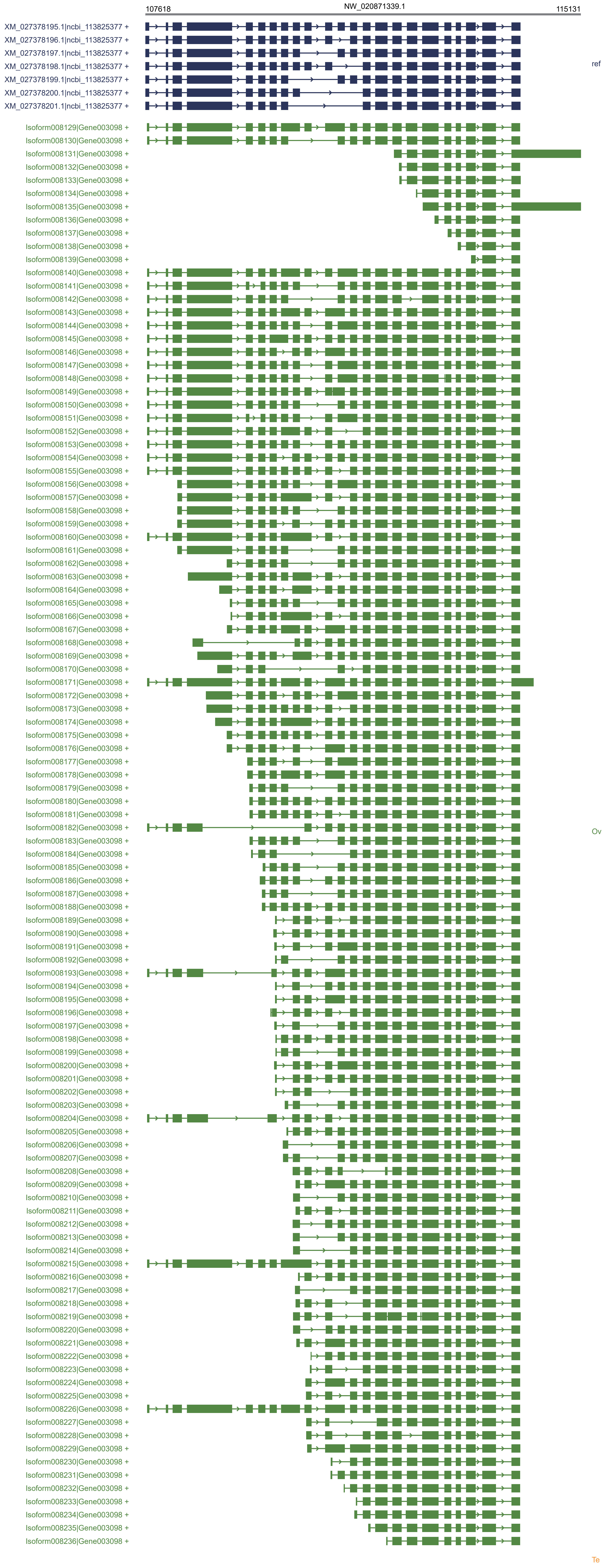

Figure S2 KEGG enrichment analysis of genes involved in AS events in testis and ovary.

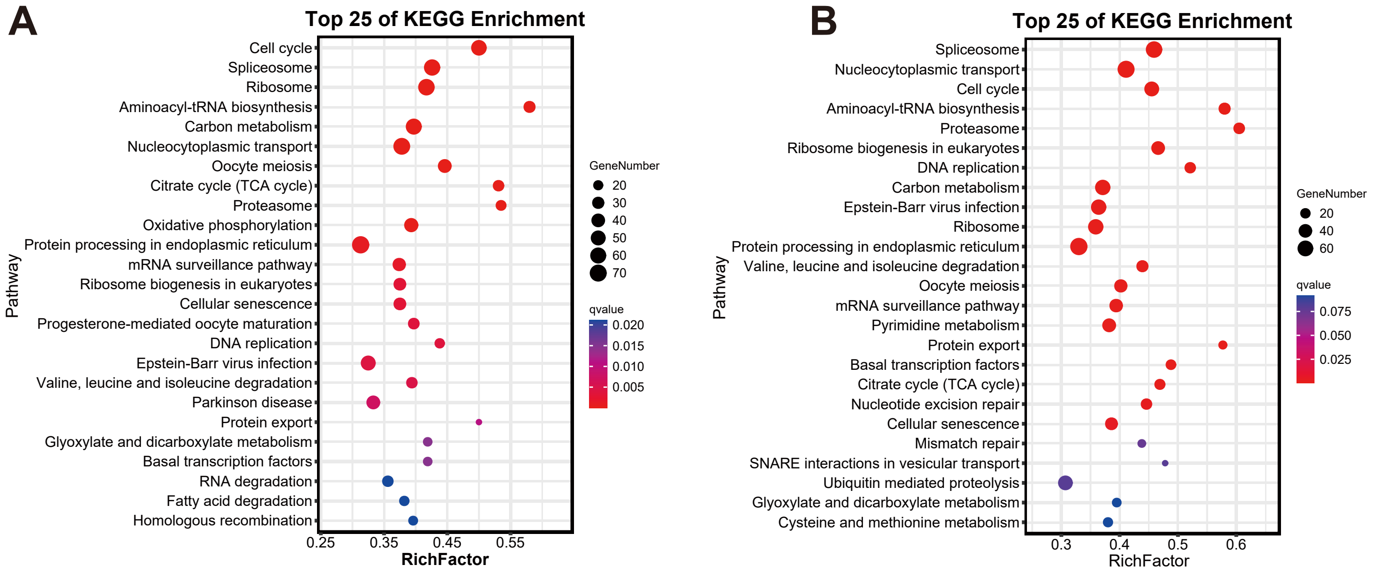

(A) KEGG pathway enriched for alternative splicing genes in ovary. (B) KEGG pathway enriched for alternative splicing genes in testis.
