# Supplementary figures and images for: Full-Length Transcriptome of Testis and Ovary Provides Insights into Alternative Splicing During Gonadal Development in Litopenaeus vannamei
Source: Int J Mol Sci. 2025 Jun 19;26(12):5863. doi: 10.3390/ijms26125863 (PMC12192809; doi:10.3390/ijms26125863)

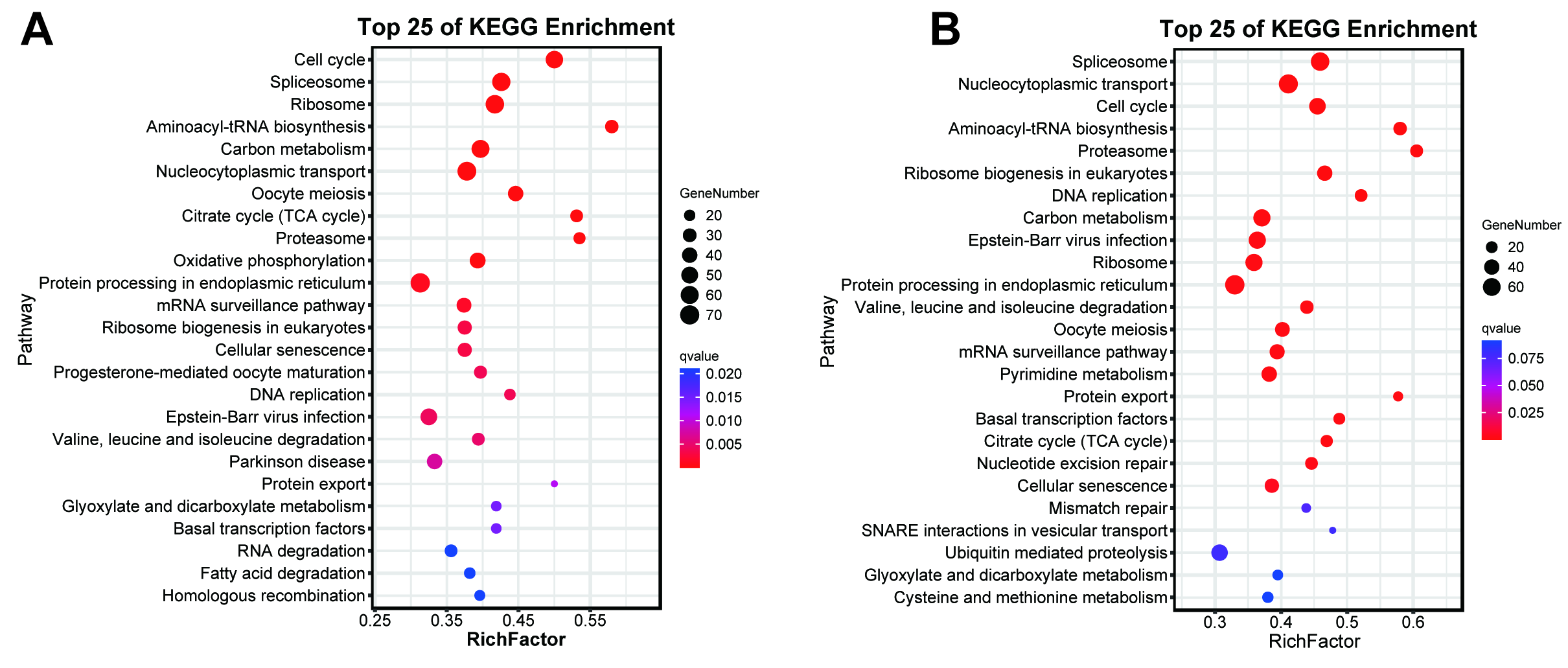

Supplement: Supplementary file 1 [file ijms-26-05863-s001.zip › Figure S2.tif]
